# Supplementary material for: Associative Learning of Quantitative Mechanosensory Stimuli in Honeybees
Source: Insects. 2024 Feb 1;15(2):94. doi: 10.3390/insects15020094 (PMC10889140; doi:10.3390/insects15020094)
Supplement: Supplementary file 1 [file insects-15-00094-s001.zip › Strelevitz_suppl_mat_corrected.pdf]

Supplementary Material

Supplementary Figure S1: Setup schematics

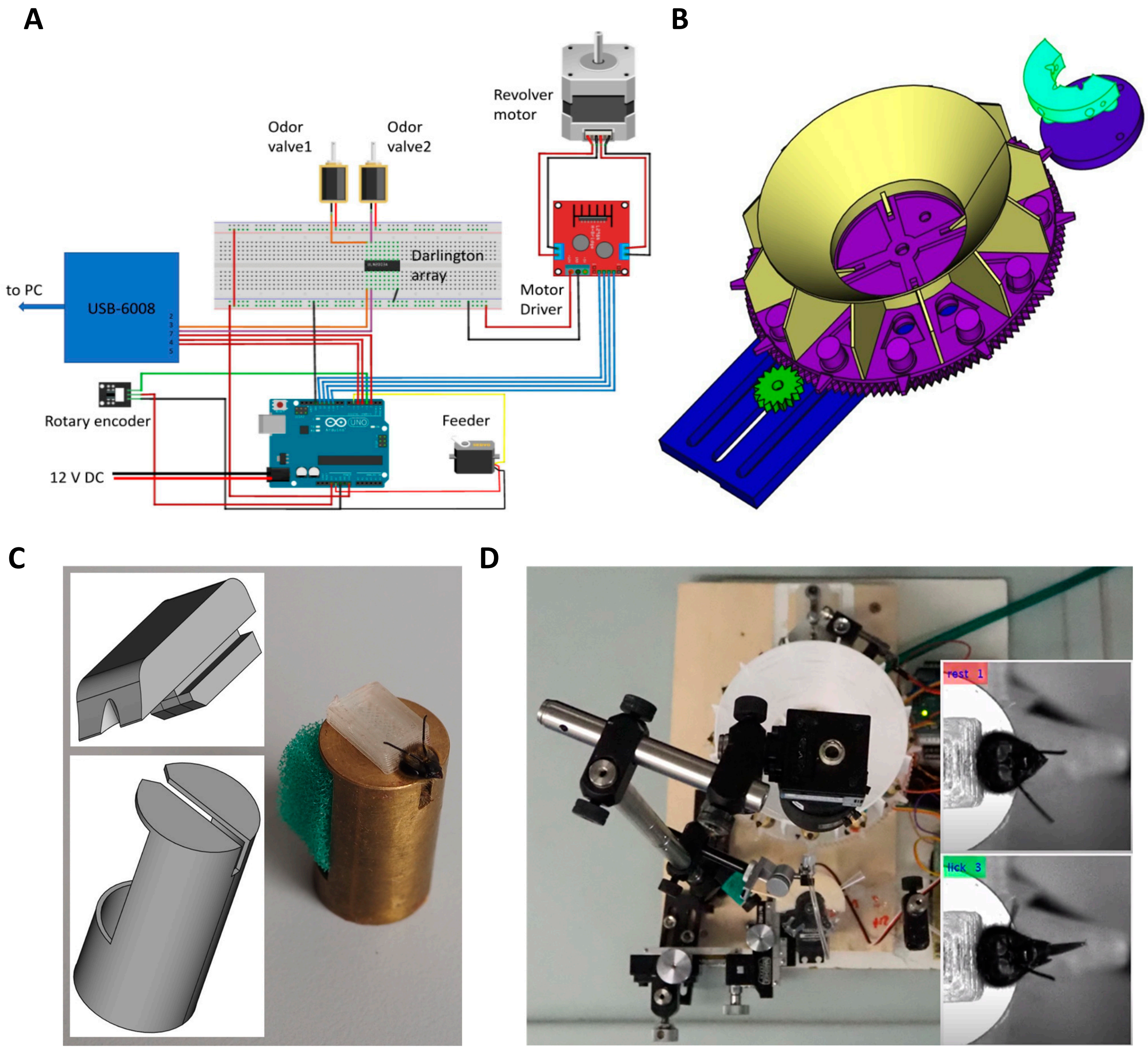

Figure S1: Setup schematics. (**A**) Electronic control circuits. (**B**) 3D scheme of the mechanical assembly. Left blue, Per_Mount; left green, MOTOR_GEAR; purple, PER_wheel; gold, cover; right green, UPPER_feeder; right blue, LOWER_feeder. (**C**) Bee mounting. Right: A bee secured in place for handling and experimentation; lower inset: scheme of the mount; upper inset: scheme of the head holder. (**D**) Camera positioning. The camera is positioned directly above the site of stimulation such that the head of a given bee is centered in the left third of the field of view. Upper inset: example of a resting state; lower inset: example of a licking state.

Supplementary Figure S2: Continuous analyses applied to the olfactory data.

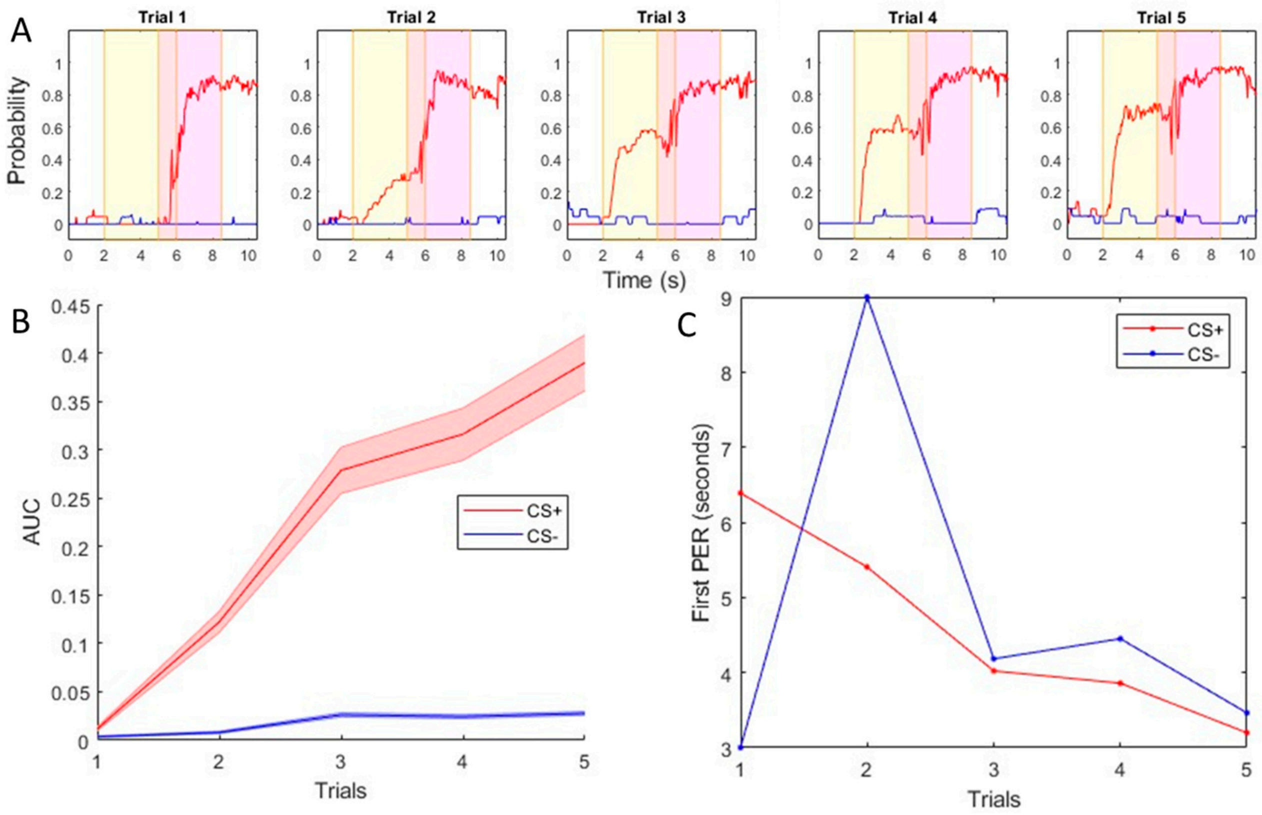

Figure S2: Continuous analyses applied to the olfactory data. (**A**) A trial-by-trial view of the probability of proboscis extension, plotted over time in seconds. Results are averaged across all bees (*n* = 24). The yellow panel indicates the time duration of the conditioned stimulus (odor) and the magenta panel is the time duration of the unconditioned stimulus (25% sucrose solution). There is a 1 second overlap. (**B**) Mean area under the probability curves for CS+ and CS- during each trial. (**C**) The mean proboscis extension latency of subjects which respond during a given trial, in seconds.

Supplementary Figure S3: Divided analysis before and after sucrose reward delivery

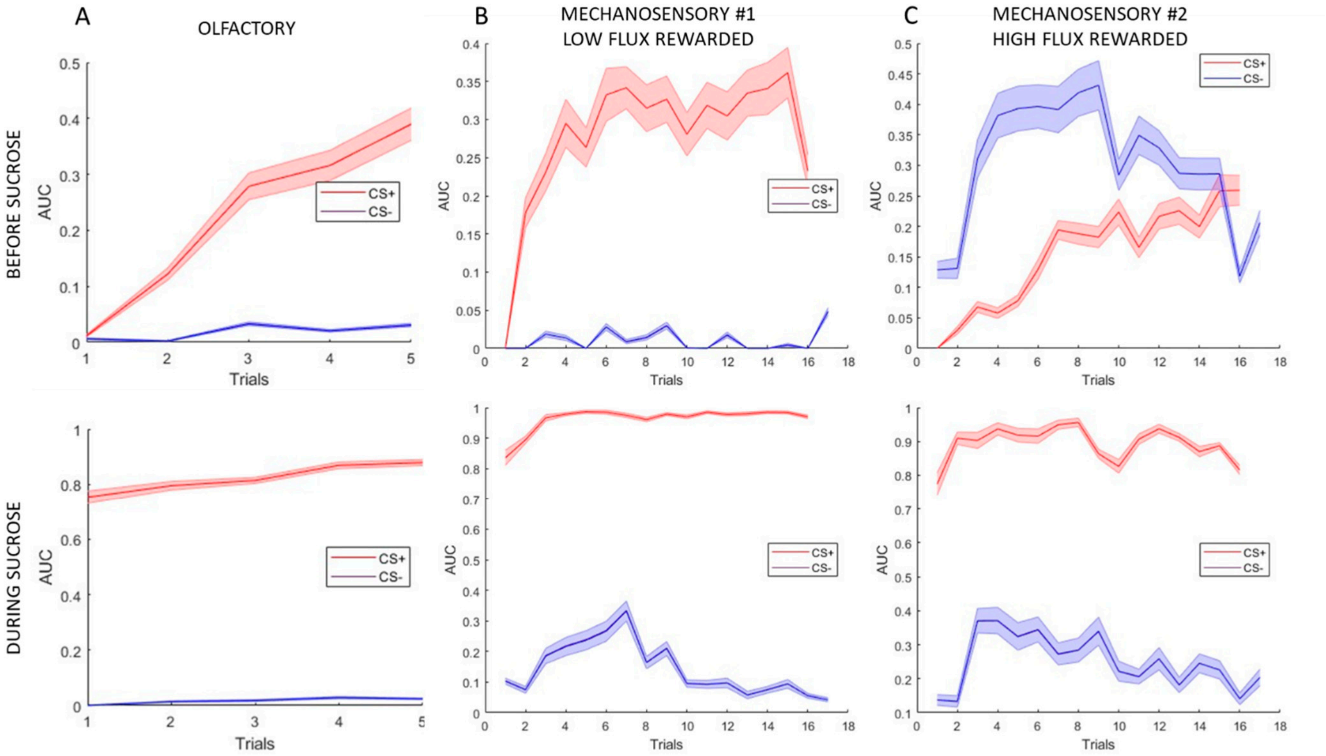

Figure S3: Mean area under the probability curves, before and after the delivery of the sucrose reward. (**A**) Data from the olfactory experiment, in Fig. 2. (**B**) Data from the first mechanosensory experiment, in Fig. 3. (**C**) Data from the second mechanosensory experiment, in Fig. 4.

Supplementary Figure S4: Experiment with 3:1 ratio between air fluxes, low flux is CS+

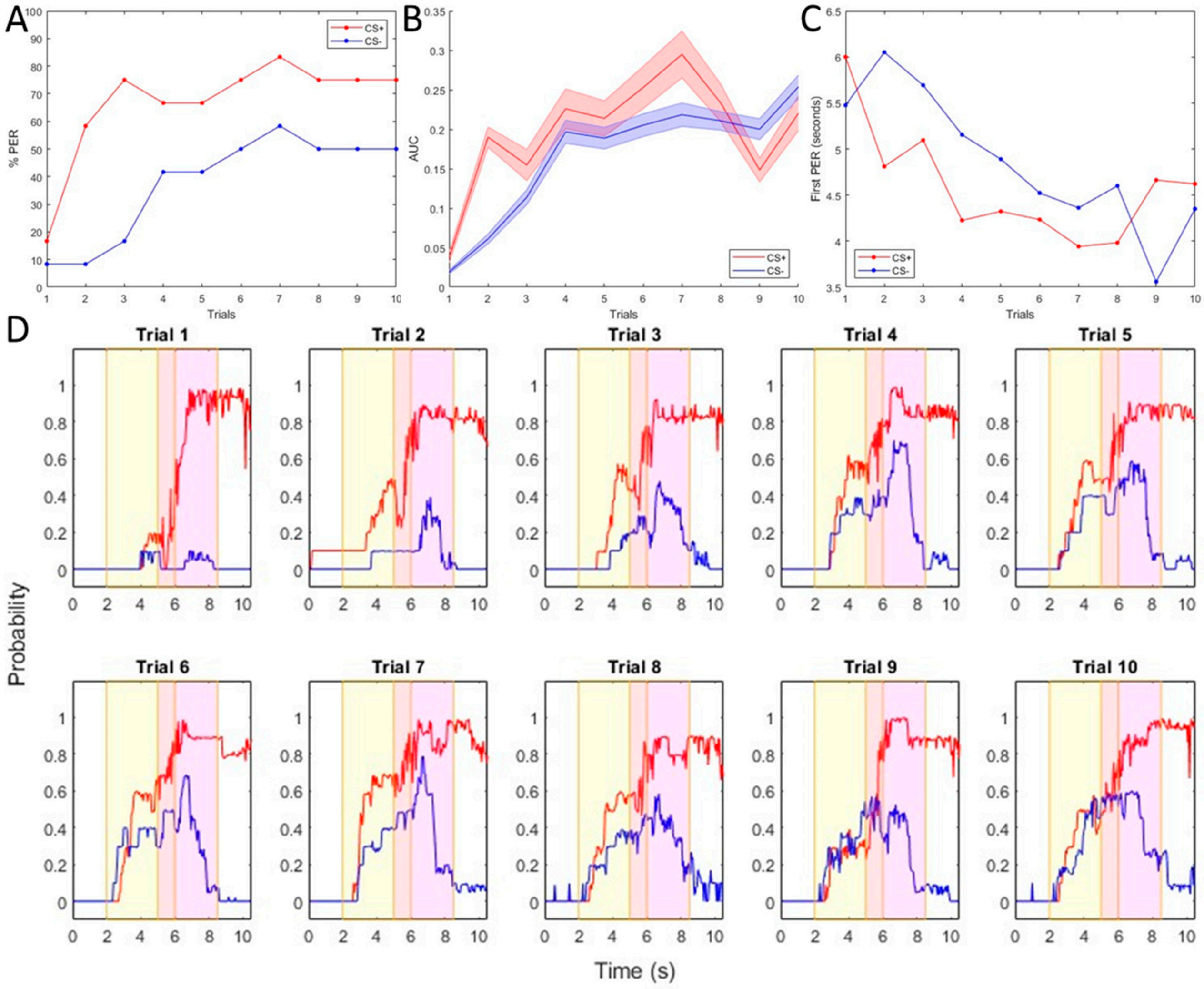

Figure S4: Mechanosensory experiment with a 3:1 ratio between air fluxes, where the lower flux is rewarded. (**A**) Classic PER response curve over trials. The low air flux stimulus (1 m/s), in red, was rewarded (CS+). The high air flux stimulus (3 m/s), in blue, was not rewarded (CS-). (**B**) Mean area under the probability curves for CS+ and CS- stimuli for each trial. (**C**) A trial-by-trial view of the results in (B), where the probability of the proboscis extension is plotted over time in seconds. Results are averaged across all bees (*n* = 12). (**D**) The mean proboscis extension latency of subjects which respond during a given trial, in seconds.

Supplementary Figure S5: Experiment with a 3:1 ratio between air fluxes, high flux is CS+

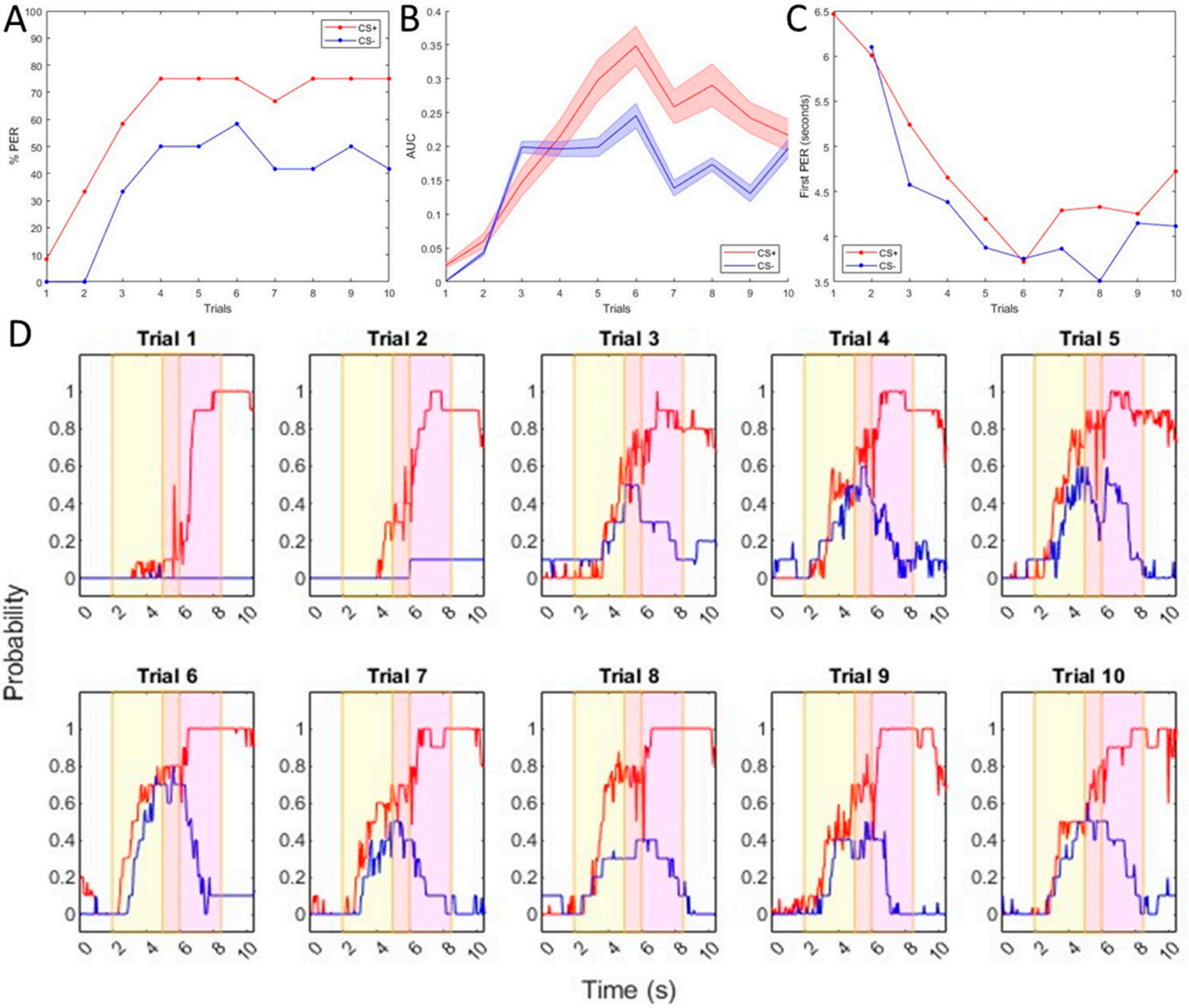

Figure S5: Mechanosensory experiment with a 3:1 ratio between air fluxes, where the higher flux is rewarded. (**A**) Classic PER response curve over trials. The high air flux stimulus (3 m/s), in red, was rewarded (CS+). The low air flux stimulus (1 m/s), in blue, was not rewarded (CS-). (**B**) Mean area under the probability curves for CS+ and CS- stimuli for each trial. (**C**) A trial-by-trial view of the results in (B), where the probability of the proboscis extension is plotted over time in seconds. Results are averaged across all bees (*n* = 12). (**D**) The mean proboscis extension latency of subjects which respond during a given trial, in seconds.

Supplementary Table S1: Statistical results for the trial-by-trial analysis of between-subject effects.

Odor conditioning

	**Trial 1**	**Trial 3**	**Trial 3**	**Trial 4**	**Trial 5**
***F*(1,130)**	0.0152	4.93	33.9	43.4	60.3
p	0.9	0.035	7.3E-08	2.6E-09	1.1E-11

Low-flux CS+

	**Trial 1**	**Trial 3**	**Trial 3**	**Trial 4**	**Trial 5**	**Trial 6**	**Trial 7**	**Trial 8**
***F*(1,130)**	0.257	2.51	1.84	3.86	2.54	4.41	5.3	6.64
p	0.61	0.13	1.90E-01	7.00E-02	1.30E-01	0.056	0.038	0.023
	**Trial 9**	**Trial 10**	**Trial 11**	**Trial 12**	**Trial 13**	**Trial 14**	**Trial 15**	**Trial 16**
***F*(1,130)**	6.17	8.69	11.2	8.44	14.9	14.9	15.7	6.84
p	0.027	0.012	0.0049	0.012	0.0012	0.0012	0.0012	0.023

High-flux CS+

	**Trial 1**	**Trial 3**	**Trial 3**	**Trial 4**	**Trial 5**	**Trial 6**	**Trial 7**	**Trial 8**
***F*(1,130)**	2.36	2.01	8.89	15.5	13	7.91	4.89	5.1
p	0.24	0.24	2.00E-02	2.60E-03	4.10E-03	0.02	0.068	0.068
	**Trial 9**	**Trial 10**	**Trial 11**	**Trial 12**	**Trial 13**	**Trial 14**	**Trial 15**	**Trial 16**
***F*(1,130)**	7.84	0.324	2.09	1.97	0.314	0.897	0.351	1.58
p	0.02	0.58	0.24	0.24	0.58	0.43	0.58	0.28

Supplementary Table S2: Mechanical part list. CAD designs of all the components of the device are provided in the FCStd format, which is a FreeCAD document. They can be opened and modified with the open source FreeCAD software. Each part can be 3D printed and some of them machined with a desktop CNC.

**File Name**	**Description**
PER_assembly_drawing	The full design containing all the mechanical components. For each part, STL files are provided as well as the full assembly for rapid 3D visualization (Fig. S1B).
per _revolver	This drawing contains multiple parts: a motor gear to be mounted on the stepper motor's shaft, the revolver, and the cover used to isolate the bees. The cover was 3D printed using PETG, whereas the other two parts can be 3D printed using any material.
PER_FRAME	These elements represent the frame onto which the stepper motor and the revolver will be mounted. The shaft can be an M5 bolt that runs through the base and the underbase. 2 bearings are placed in the slots in the center of these 2 last pieces. The base and the underbase can be joined by means of 3 bolts, after drilling and threading of correspondent holes in the base.
feeder	These are two pieces: the servo_ mount which is fixed onto the servo motor shaft and the feeder_mount which is connected to the servo mount with three rods of 4 mm in diameter. The distance between the two parts should be adjusted to fit the whole structure.
head_holder	The piece used for fixing in place the head of the bee. It should be 3D printed using a soft polymer like TPU.
bee_MOUNT	The piece where bees are fixed for the experiment.

Supplementary Table S3: Electronic component list.

component	function	producer code
stepper motor driver	drive revolver motor	Duokon L298N Dual H Bridge DC stepper Motor Drive Module
rotary encoder	positioning of the revolver	Hailege LM393 Photoelectric Sensor/Count Sensor
servo motor	feeder movements	Parallax Standard Servo 900-00005
stepper motor	rotate the revolver	Wantai NEMA17 42BYGHW811
Arduino board	microcontroller	Arduino Uno
USB NI DAQ board	communication between the PC and the PER hardware	National Instruments USB-6008
Darlington transistor array	power the actuators	STMicroelectronics ULN2003
flux-controlled valve	control air flux	Camozzi AP-621L-LR3-GPH
camera	recording proboscis motion	Imagingsource DMK27BUR0135
lens	imaging experiental area on camera chip	Tamron M13VG550, 5.0-50 mm F/1.4 1/3 CS

Supplementary Video S1: Experimental sequence.

A video showing the camera's recording of a single trial. After 2 seconds the CS+ (an odor, in this case 1-hexanol) is applied, and after 5 seconds the US is added, by first touching the antennae with a sucrose-soaked stick and then providing sucrose solution from the feeder. The inset shows the time in seconds and its colors indicate the result of the machine-learning classification of whether the proboscis is extended or not (lick/rest).
